# Assessment of Pain and Quality of Life in Lichtenstein Hernia Repair Using a New Monofilament PTFE Mesh: Comparison of Suture vs. Fibrin-Sealant Mesh Fixation

**DOI:** 10.3389/fsurg.2014.00045

**Published:** 2014-11-28

**Authors:** René H. Fortelny, Alexander H. Petter-Puchner, Heinz Redl, Christopher May, Wolfgang Pospischil, Karl Glaser

**Affiliations:** ^1^Department of General, Visceral and Oncological Surgery, Wilhelminenspital, Vienna, Austria; ^2^Paracelsus Private Medical University, Salzburg, Austria; ^3^Ludwig Boltzmann Institute for Experimental and Clinical Traumatology, Austrian Cluster for Tissue Regeneration, Vienna, Austria; ^4^Trauma Surgery, Landeskrankenhaus St. Pölten, St. Pölten, Austria

**Keywords:** monofilament PTFE, Lichtenstein hernia repair, fibrin glue, postoperative pain, quality of life

## Abstract

**Background:** Inguinal hernia repair is one of the most common operations in general surgery. The Lichtenstein tension-free operation has become the gold standard in open inguinal hernia repair. Despite the low recurrence rates, pain and discomfort remain a problem for a large number of patients. The aim of this study was to compare suture fixation vs. fibrin sealing by using a new monofilament PTFE mesh, i.e., the Infinit^®^ mesh by W. L. Gore & Associates.

**Methods:** This study was designed as a controlled prospective single-center two-cohort study. A total of 38 patients were enrolled and operated in Lichtenstein technique either standard suture mesh fixation or fibrin-sealant mesh fixation were used as described in the TIMELI trial. Primary outcome parameters were postoperative complications with the new mesh (i.e., seroma, infection), pain, and quality of life evaluated by the VAS and the SF-36 questionnaire. Secondary outcome was recurrence assessed by ultrasound and physical examination. Follow-up time was 1 year.

**Results:** Significantly, less postoperative pain was reported in the fibrin-sealant group compared to the suture group at 6 weeks (*P* = 0.035), 6 months (*P* = 0.023), and 1 year (*P* = 0.011) postoperatively. Additionally, trends toward a higher postoperative quality of life, a faster surgical procedure, and a shorter hospital stay were seen in the fibrin-sealant group.

**Conclusion:** Fibrin-sealant mesh fixation in Lichtenstein hernioplasty effectively reduces acute and chronic postoperative pain. Monofilament, macro-porous, knitted PTFE meshes seem to be a practicable alternative to commonly used polypropylene meshes in open inguinal hernia repair.

## Introduction

Inguinal hernia repair is one of the most common surgical operations in general surgery. In 2010, 19,515 inguinal hernias were treated in Austria, 17,094 among men and 2,421 among women (1). Tension-free mesh augmented operation has become the standard technique in inguinal hernia surgery (2–5). The Lichtenstein repair utilizing prosthetic meshes is the most commonly used technique. In the past, clinical trials regarding hernia repair, concentrated on the long-term analysis of recurrence rates. More recently, the focus of several clinical trials has been placed on aspects of quality of life and chronic pain syndromes (6–10). Indeed, despite the low recurrence rates [fewer than 5% (4, 11, 12)] of the Lichtenstein open tension-free mesh augmented repair postoperative pain and chronic postoperative pain syndromes still remain a problem (13–18). Moreover patients frequently report a feeling of numbness, stiffness, or foreign body, after implantation of a commonly used polypropylene mesh. Several studies and randomized clinical trials indicate that up to 30% of patients report some form of pain 1 year after Lichtenstein hernia repair (5, 19). As a result of these findings, numerous studies were initiated to estimate the ideal material for implantation in the inguinal region (20–22). There is evidence that reduced weight macro-porous meshes are associated with more patient comfort, a better physical function and less pain during activity (6, 20, 21, 23). It was demonstrated that fibrin-sealant mesh fixation is superior to suture fixation on aspects of quality of life, patient comfort, postoperative pain, and chronic pain (6, 10, 24–28). In the past, polytetrafluorethylene (PTFE) meshes were processed to micro-porous patches. The combination of hydrophobic PTFE with micro-pores led to frequent mesh graft infections, resulting in obligatory mesh explanations (22, 29, 30). The new monofilament, macro-porous, knitted PTFE mesh (Inifnit^®^ mesh; W. L. Gore & Associates) used in this study, promised to profit from the advantages of PTFE, such as low foreign body reaction, without having to deal with the frequent complication of mesh graft infection. Especially in an experimental study, the advantages of condensed PTFE meshes in combination of fibrin sealant for fixation could be demonstrated (31).

## Materials and Methods

This study was a controlled, unicentric, two-cohort pilot study at the Department of General, Visceral and Oncological Surgery, Wilhelminenspital Vienna and designed as a pilot study for a further planned randomized controlled trial study. The permission of the local ethical committee was granted. All patients seen at the outpatient ward for hernia diseases at the Department of General, Visceral and Oncologic Surgery, Wilhelminenspital Vienna, with the diagnosis of a unilateral primary inguinal hernia were considered for entry into the study. All enrolled patients had to fulfill the inclusion criteria and had to give informed consent to participate in this study. Patients were randomized using an online randomizing tool. Patients eligible for inclusion were aged between 18 and 80 years; had a unilateral primary inguinal hernia; were scheduled for elective operation and had the intellectual capacity to participate in this study. Exclusion criteria were femoral hernias; recurrent inguinal hernias; American society of Anesthesiologists (ASA) IV or higher; a poor understanding of the German language; non-compliance; incarcerated or strangulated hernias; and pregnancy. A total of 38 patients with the diagnosis of a unilateral primary inguinal hernia were enrolled, 20 patients were assigned to the suture group (SUT) and 18 patients were assigned to the fibrin-sealant-group (FS). In the SUT cohort, the mesh was fixated only with sutures and in the second cohort the mesh was fixated with fibrin glue, whereas one suture was allowed to recreate a new inguinal ring by suturing the two crossed tails of the mesh lateral to spermatic cord. Originally, it was planned to enroll a total of 50 patients, 25 patients for each group. However, W. L. Gore & Associates unexpectedly stopped the sale of the Infinit mesh, without giving reason. This forced our study group to stop the patient enrollment with a total of 38 patients, 20 patients in the SUT group, and 18 in the FS group (Table 1). Patients were allocated to one of the both groups by the study group. The study group monitored the equality of the two groups regarding age, sex, pain, hernia size et cetera. Follow-up period was 1 year. We exclusively used the Infinit^®^ mesh, W. L. Gore & Associates a monofilament, macro-porous, knitted, middle-weight, and polytetrafluoroethylene (PTFE) surgical mesh (weight 70 g/m^2^; pore size is 2–3 mm and the thickness of the monofilament fibers is 0.4 mm).

**Table 1:** **An overview of patients characteristics and distribution of different hernia types according to the Aachen classification**.

	FS group	SUT group	Total
Patients	18	20	38
Males	16	18	34
Females	2	2	4
Age	47.28 ± 14.93	54.35 ± 16.68	51 ± 16.06
BMI	25.06 ± 4.1	25.88 ± 3.2	25.49 ± 3.63
Aachen classification
L I	0	6	6
L II	8	6	14
L III	4	1	5
M I	4	0	4
M II	0	6	6
Mc I	1	0	1
Mc II	1	1	2

### Operating procedures

All operations were performed applying general anesthesia. Thirty minutes before the end of the operation, 1 g Paracetamol was administered intravenously to each patient. At the end of the operation, the OP-site was infiltrated with Naropin. Antibiotic prophylaxis was sustained by the administration of a single shot of second, respectively third generation cephalosporines during the operation. To treat postoperative pain, no NSAIDs or COX-2 inhibitors but paracetamol and tramadol were used.

### Suture fixation

All surgeons in the study group adhered to the refined standard Lichtenstein open tension-free hernioplasty technique as described by Amid (4).

### Fibrin-sealant mesh fixation

The skin incision and the preparation of the inguinal region in the fibrin-sealant group did not differ from that applied in the SUT group. The preliminary shaping of the mesh was identical to that described for the suture group. A single absorbable suture was used in the fibrin-sealant group to narrow the internal inguinal ring and fixate the two tails of the mesh lateral to the internal ring (32). However, the mesh was then exclusively fixated with 2 ml of Fibrin glue (500 IU Tissucol^®^ Baxter Healthcare). The fibrin glue was prepared according to the manufacture’s instruction, and applied using the Duploject syringe and a spray head (32). After the preparation of the inguinal region and correct positioning of the mesh the complete prepared Tissucol was placed over the entire surface of the mesh, using the spray-effect. Subsequent, the mesh was pressed onto the tissue for 2 min until the polymerization of the glue was completed (32). Closure of the external oblique aponeurosis and the skin was identically to that applied in the suture group.

### Pain VAS score

The visual analog scale (VAS) consists of a simple scale with a length of exactly 100 mm, on which patients were asked to rate their pain from 0 (completely absence of pain) to 100 (worst imaginable pain) (33).

All enrolled patients were asked to mark their current sensation of pain on a VAS preoperatively; the first day postoperatively; the second day postoperatively; at the day of discharge; 6 weeks postoperatively, 6 months postoperatively, and 1 year postoperatively.

### Quality of Life – Short-form 36 questionnaire

The short-form 36 (SF-36) is a short-form health survey with 36 questions. It yields an 8-scale profile of functional health and well-being scores. The eight scales are physical functioning (PF), role-physical (RP), bodily pain (BP), general health (GH), vitality (VT), social functioning (SF), role-emotional (RE), and mental health (MH).

These eight scales are additionally summarized psychometrically to two summary measures, physical health (PHS), and mental health (MHS). The evaluation of the SF-36 survey is performed by standardized, automatic computer programs. Thus for each scale and both summary measures, a score from 0 (worst) to 100 (best) will be generated.

All patients were asked to complete the SF-36 questionnaire preoperatively, 6 weeks after the operation, 6 months after the operation, and 1 year after the operation.

### Statistical analysis

All statistical analyses were performed with the SPSS 17 software. Primary outcome parameters were pain, quality of life, and post-OP complications. Secondary outcome parameter was recurrence.

The mean, SD, median, minimum, maximum, and quartiles of the VAS and SF-36 scores were calculated per cohort (preoperatively, 6 weeks postoperatively and 1 year postoperatively). An unpaired *t*-test was performed to investigate the discrepancy among the two groups (fibrin-sealant mesh fixation versus suture mesh fixation) regarding the mean difference between the pre- and 6 weeks postoperative, the mean difference between the pre- and 6 months SF-36 scores, as well as the mean difference between the pre- and 12 months postoperative SF-36 scores for each cohort. To compare and analyze the kind and frequency of complications, i.e., seroma or infection, among the two groups (fibrin-sealant mesh fixation versus suture mesh fixation) all complications were recorded and described in each group.

Originally, it was intended to include 100 patients (*n* = 50 per group). Accordingly, the sample size was calculated with Russ Length’s online sample size calculator (recommended by the department of medical statistics; Medical University Vienna). A sample size of 50 individuals was calculated, utilizing an unpaired *t*-test. The primary outcome parameter “pain” (evaluated by the VAS) was considered for the calculations. A two-sided significance of 0.05 and a power of 0.80 were applied. As the endpoint of the study was not met, this power analysis was irrelevant. Results of the patients who could finally be included were tested for normal distribution and a Kolmogorov–Smirnov test was applied. In this test, no significance was achieved, thus normal distribution was assumed.

## Results

Because of the premature end of the study, statistical power was again verified for the obtained set of data.

### Study population

A total of 38 patients with the diagnosis of a unilateral primary inguinal hernia were enrolled in this study, 20 patients were assigned to the suture group and 18 patients were assigned to the fibrin-sealant group. The two groups were comparable concerning all study variables except the mean age; however, the difference of the age was none significant.

### Operation time

Allover, the mean duration of the surgical procedure was 52 ± 20.12 min (range 25–123). Within the suture group, the mean duration of the surgical procedure was 53.3 ± 24.66 min (range 25–123). Within the fibrin-sealant group, the mean duration of the surgical procedure was 50.56 ± 14.06 min (range 35–90).

### Hospital stay

The hospital stay of all enrolled patients revealed a mean of 4 days (SD 1.09; range 2–7). Twenty patients assigned to the suture group stayed for a mean of 4.15 days (SD 0.88; range 2–5) and 18 patients assigned to the fibrin-sealant group stayed for a mean of 3.83 days (SD 1.3; range 2–7), respectively at the in-patient ward.

### Complications

In two patients (5.3%), a partial resection of the iliohypogastric nerve was performed consequently to intra-operative damage (both were assigned to the fibrin-sealant group). No further intra-operative complications were reported.

Seven (18.4%) of all patients participating in this study were suffering from postoperative complications. Six (15.8%) were suffering from a hematoma and one (2.6%) patient was suffering from a seroma. Within the suture group four (20%) patients were suffering from a hematoma and none from a seroma; whereas two (11.1%) patients within the fibrin-sealant group were suffering from a hematoma and one (5.6%) patient from a seroma. There was no significant difference concerning postoperative surgical complications, between the two groups. None of the patients suffering from postoperative complications needed any further clinical treatment.

One recurrent hernia in the fibrin-sealant group was detected by ultrasound 1 year after the operation. In this patient, the recurrent hernia was treated by a laparoscopic transabdominal preperitoneal (TAPP) procedure.

### Pain

Preoperative pain assessed by the VAS, was comparable between the two groups 25.83 ± 17.34 (range 0–70) within the suture group and 24.06 ± 15.3 (range 0–75) within the fibrin-sealant group. Early postoperative pain assessed at rest on day 1, day 2, and on the day of discharge, was not significantly different between the two groups.

On re-evaluation after 6 weeks, the mean VAS score of 8.61 ± 10.39 of the patients within the fibrin-sealant group was significantly lower than the mean VAS score of 19.72 ± 18.98 of the patients within the suture group *P* = 0.035.

On re-evaluation after 6 months, the mean VAS score of 6.82 ± 9.72 of the patients within the fibrin-sealant group was significantly lower than the mean VAS score of 17.61 ± 16.41 of the patients within the suture group *P* = 0.023.

At the final assessment after 1 year, the mean VAS score of 5.93 ± 8.06 of the patients within the fibrin-sealant group was significantly lower than the mean VAS score of 18.17 ± 17.52 of the patients within the suture group *P* = 0.011.

Additionally, the number of patients suffering from moderate-severe pain (VAS ≥30) 1 year postoperatively was lower in the fibrin-sealant group 1/18 (0.55%) versus 5/20 (25%) in the suture group.

**Figure 1:**
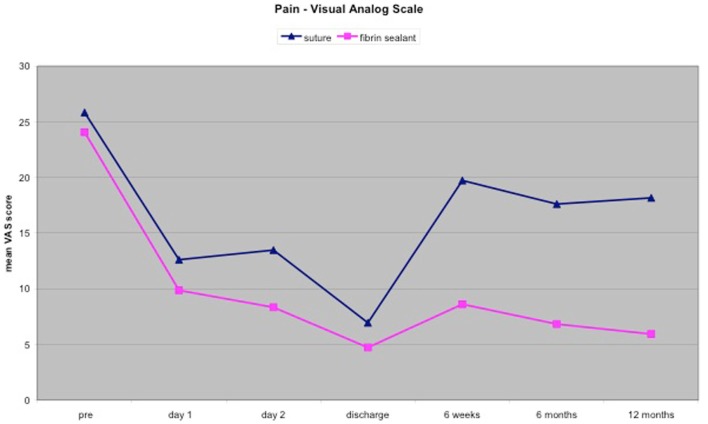
**Change of the mean VAS score within the two groups over time**.

The mean VAS scores of both groups decreased rapidly after the operation and during the hospital stay to a bottom level at the day of discharge from hospital. On re-evaluation after 6 weeks, the mean VAS scores of both groups increased from the bottom level seen at the day of discharge from hospital (see Figure 1). However, the mean VAS score of the suture group increased to a significant higher level than the mean VAS score of the fibrin-sealant group. During the period between the re-evaluation after 6 weeks and the re-evaluation after 6 months the mean VAS scores of both groups decreased, whereas the mean VAS score of the fibrin-sealant group was significantly lower compared to the mean VAS score of the suture group. During the period between the re-evaluation after 6 months and the final assessment after 1 year, the mean VAS score of the suture group increased in contrast to the mean VAS score of the fibrin-sealant group, which decreased during that period, to a significant lower level compared to the suture group.

### Quality of life

**Figure 2:**
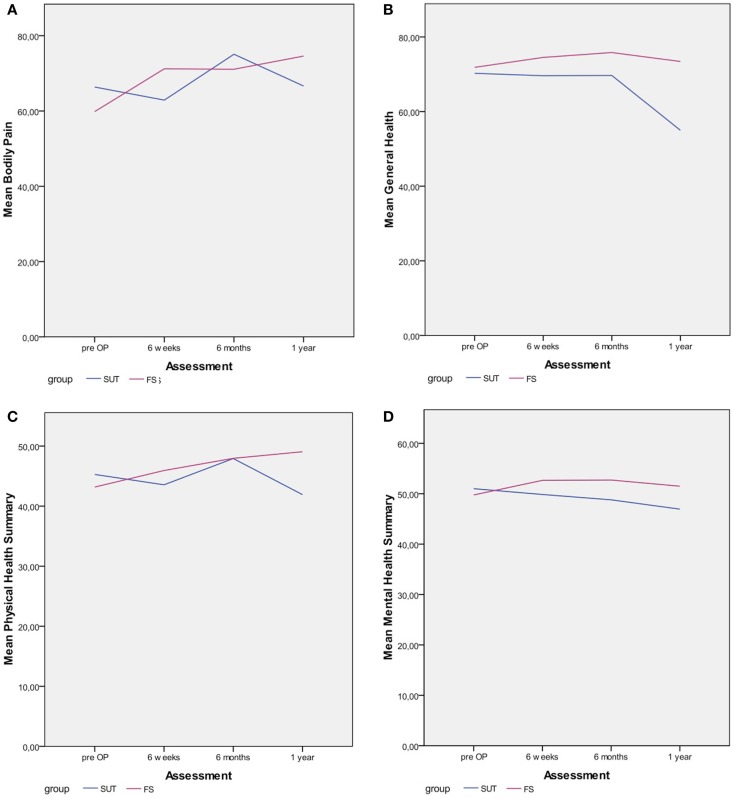
**Differences between group in the most relevant SF-36 outcome parameters**.

In both groups, the mean scores of the PF scale increased constantly during the follow-up period. In the suture group, the mean score increased from 69.38 ± 26.15 to 80.08 ± 24.73 1 year after the operation and in the fibrin-sealant group the mean scores increased from 70 ± 19.93 to 79.53 ± 21.71 1 year after the operation. The mean scores of the RP scale was constantly increasing in the fibrin-sealant group from 55.77 ± 37.53 to 81.62 ± 30.78 1 year after the operation, whereas the mean score in suture group decreased 6 weeks after the operation to a bottom level of 51.18 ± 42.52. From this bottom level after 6 weeks, the mean score of the RP scale in the suture group increased to 68.68 ± 39.67 1 year after the operation. In the fibrin-sealant group, the mean score of the BP scale (Figure 2A) immediately raised from 59.85 ± 18.77 to 71.21 ± 25.12 6 weeks after the operation. During the period from 6 weeks and 6 months postoperatively, the mean score of the BP scale did not significantly change to 71.10 ± 25.11. From the assessment 6 months after the operation, the mean score of the BP scale in the fibrin-sealant group, raised to 74.60 ± 21.09 1 year postoperatively. In the suture group, the mean score of the BP scale immediately felt in contrast to the fibrin-sealant group, from 66.38 ± 20.21 to 62.89 ± 27.75 6 weeks postoperatively. From the level at 6 weeks postoperatively, the mean score of the BP scale raised to 75.09 ± 24.36, to finally fall to 66.63 ± 34.59 1 year after the operation. Thus, the mean score of the BP scale in the fibrin-sealant group clearly improved from 59.85 ± 18.77 preoperatively to 74.60 ± 21.09 1 year postoperatively; in contrast to the mean score of the BP scale in the suture group, which did not significantly improve from 66.38 ± 20.21 preoperatively to 66.63 ± 34.59 1 year postoperatively. In the fibrin-sealant group, the mean GH score (Figure 2B) increased from 71.85 ± 7.98 preoperatively to 74.54 ± 13.29 6 weeks postoperatively and further to 75.82 ± 11.10 6 months postoperatively. From the 6 months level, the mean GH score decreased to 73.43 ± 13.34 1 year postoperatively. In the suture group, the mean GH score immediately decreased from 70.25 ± 16.74 preoperatively to 69.58 ± 19.55 6 weeks postoperatively. Then in the period from 6 weeks to 6 months postoperatively, the mean GH score did not change significantly to 69.68 ± 20.86. However, the mean GH score then clearly decreased to its lowest level of 54.98 ± 28.37 1 year postoperatively. One year postoperatively, the mean GH score of 73.43 ± 13.34 in the fibrin-sealant group was significantly higher (*P* = 0.015) than the mean GH score of 54.98 ± 28.37 in the suture group.

In the fibrin-sealant group, the mean VT score instantly raised from 55.38 ± 11.82 preoperatively to 65.54 ± 19.97 6 weeks postoperatively and further to 66.65 ± 19.15 6 months postoperatively. Subsequently, the mean VT score in the fibrin-sealant group did not significantly change to 66.10 ± 20.10 1 year postoperatively. In contrast to the fibrin-sealant group, the mean VT score in the suture group instantly felt from 65.31 ± 21.18 preoperatively to 58.04 ± 21.96 6 weeks postoperatively. The mean VT score then rose to 69.68 ± 20.86 6 months postoperatively, to finally fall to its lowest level of 52.54 ± 29.54 1 year postoperatively. The mean SF score in the fibrin-sealant group increased from 75.00 ± 18.19 preoperatively to 78.27 ± 26.61 6 weeks postoperatively and furthermore to 87.30 ± 17.63 6 months postoperatively. From the level at 6 months postoperatively, the mean SF score decreased to 83.82 ± 19.95 1 year postoperatively. In contrast to the fibrin-sealant group, the mean SF score in the suture group instantly decreased postoperatively from 83.59 ± 20.96 preoperatively to 78.23 ± 26.85 6 weeks postoperatively. Subsequently, the mean SF score increased to 81.98 ± 23.79 6 months postoperatively. However, the mean SF score then decreased again from the level at 6 months postoperatively to 68.23 ± 37.95 1 year postoperatively. In the fibrin-sealant group, the mean RE score advanced from 69.23 ± 35.17 preoperatively to 85.51 ± 30.18 6 weeks postoperatively. Subsequently, the mean RE score in the fibrin-sealant group did not significantly change during the remaining follow-up period (83.66 ± 30.27 6 months postoperatively and 85.51 ± 30.18 1 year postoperatively). In contrast, the mean RE score in the suture group immediately regressed postoperatively from 75.00 ± 35.05 preoperatively to 70.09 ± 41.74 6 weeks postoperatively. The mean RE score continued to regress to 68.43 ± 43.90 6 months postoperatively and further to 50.09 ± 46.55 1 year postoperatively. One year postoperatively, the mean RE score of 85.51 ± 30.18 in the fibrin-sealant group was significantly higher (*P* = 0.08) than the mean RE score of 50.09 ± 46.55 in the suture group. The mean MH score in the fibrin-sealant group did not significantly change during the entire follow-up. Likewise in the fibrin-sealant group, the mean MH score in the suture group did not significantly change in the first 6 months postoperatively. However, the mean MH score in the suture group then clearly decreased from 71.98 ± 18.08 at 6 months postoperatively to 60.78 ± 32.50 1 year postoperatively. In the fibrin-sealant group, the mean physical health summary (PHS) score (Figure 2C) improved constantly from 43.17 ± 8.59 preoperatively to 49.05 ± 8.65 1 year postoperatively. In contrast to the fibrin-sealant group, the mean PHS score in the suture group deteriorated from primarily 45.27 ± 8.01 preoperatively to 43.55 ± 9.64 6 weeks postoperatively. The mean PHS score then improved to 47.92 ± 8.56 1 year postoperatively. However, the mean PHS score in the suture group subsequently decreased to 41.91 ± 19.54 1 year postoperatively. In the fibrin-sealant group, the mean MHS score (Figure 2D) did not significantly change during the entire follow-up period Whereas, the mean MHS score in the suture group decreased constantly from 51.00 ± 7.79 preoperatively to 46.94 ± 12.18 1 year postoperatively.

## Discussion

Since the routine utilization of prosthetic meshes in inguinal hernia surgery has become popular and because of the thereby obtained low recurrence rates, most novel clinical trials focus on aspects of quality of life and postoperative pain. More recently several trials indicated that fibrin-sealant mesh fixation is superior to suture fixation on aspects of quality of life, postoperative pain, and patient comfort (6, 24–27, 32). However, the data received from these trials is still limited and therefore need further investigations.

Besides the fixation of the mesh, the mesh itself frequently is the focus of observation. In this study, the Inifnit^®^ mesh by W. L. Gore & Associates, a new monofilament, macro-porous, knitted PTFE mesh, was used. As a result of the arbitrary withdrawal from market of the Inifnit^®^ mesh by W. L. Gore & Associates without any adverse events associated with the mesh; this study was constrained to abort prematurely. However, Inifnit^®^ mesh was well tolerated in all enrolled patients and low complication rates were recorded. Therefore, a monofilament, macro-porous, and knitted PTFE mesh seems to be a practicable alternative to common standard polypropylene meshes in Lichtenstein hernioplasty.

This study further indicates that fibrin-sealant mesh fixation results in a higher postoperative quality of life and in significantly less postoperative pain compared to suture mesh fixation. Though it must be noted that the number of patients included in this study was limited to 38 patients and therefore all statistic analyses have to be evaluated with caution. Especially, the analysis of the SF-36 questionnaire demands a higher number of patients to obtain reliable and significant results. The results of the analysis of the VAS scores appeared more reliable, related to the fact that this study was powered for this endpoint. Accordingly, the difference of the postoperative pain (6 weeks, 6 months, and 1 year postoperatively) between both groups was significant in favor of the fibrin-sealant group. Prior to this study, the maximum difference between the parameters pain and quality of life was expected in the early postoperative period, due to the traumatic suture fixation compared to the atraumatic fibrin-sealant mesh fixation. The maximum difference between the parameters pain and quality of life were though at the end of the follow-up in this study, 1 year postoperatively. The fact that fibrin glue is biodegradable, biocompatible, and is replaced by connective tissue within 2 weeks might play a major role, since non-absorbable sutures are not biodegradable and potentially maintain a chronic inflammatory process associated with pain and discomfort. These results were reflected by the TIMELI trial, as in the TIMELI trial the difference of postoperative pain between both groups was significant in favor of the fibrin-sealant group 1 and 6 months postoperatively (32). Other surgical complications beside postoperative pain were distributed equally among both groups in this study. The novel composite primary study endpoint suggested in the TIMELI trial [the incidence of at least one moderate-severe (VAS ≥30) complication regarding pain and/or numbness and/or groin discomfort] is an interesting approach to provide a combined quantitative assessment of these parameters. Although we would propose to summate all three values and thus, receiving an overall measure ranging from 0 to 300. This would allow identifying the level of impairment of the postoperative quality of life regarding pain, numbness, and groin discomfort.

In addition to lower postoperative pain and a superior postoperative quality of life detected in the fibrin-sealant group, this study demonstrates another benefit of the fibrin-sealant mesh fixation. Namely, the accelerated surgical procedure, this study indicates that the operation is shorter when fibrin glue instead of sutures is used to fixate the mesh. This effect is expected to enlarge when fibrin-sealant mesh fixation has become routine and the respective surgeons are acquaint with the technique, since there is a learning curve applying fibrin glue. Furthermore the mean hospital stay of the patients assigned to the fibrin-sealant group was shorter compared to the mean hospital stay of the patients in the suture group. However, the shortened operation in the fibrin-sealant group was not significant but other similar trials especially the TIMELI trial did reflect this finding (32).

Among surgeons, there is always controversy about the recurrence rates after fibrin-sealant mesh fixation. Many surgeons fear that the enhanced postoperative quality of life and the low postoperative pain after fibrin-sealant mesh fixation is achieved at the expense of the low recurrence rates known after standard suture mesh fixation. In this study, one recurrent hernia was found in the fibrin-sealant group and none in the suture group, though the limited number of patients in this study that it is not possible to draw a conclusion. Although the TIMELI trial demonstrated very low recurrence rates for both suture and fibrin-sealant mesh fixation (32).

Overall, the results of this study support the findings of similar studies (6, 24–27, 32) and further underline the benefits of fibrin-sealant mesh fixation on aspects of pain, quality of life, the duration of the surgical procedure, and the duration of the hospital stay.

For future trials, we suggest to choose a longer follow-up period and a lager number of patients, to receive more reliable recurrence rates. New questionnaires more specific for the surgical problem, which have not been available when the study was conducted could also be implemented in future trials (e.g., the App provided by Carolinas Medical Center). Additionally further investigations, whether large medial hernias (M III, Aachen classification) are capable for fibrin-sealant mesh fixation, have to be made since M III hernias were excluded in this study as well as in similar studies.

## Conclusion

In conclusion, this study demonstrates that fibrin-sealant mesh fixation is well tolerated and effective in reducing postoperative pain and improving postoperative quality of life. Therefore, fibrin-sealant mesh fixation represents an excellent alternative to standard suture mesh fixation in Lichtenstein hernioplasty.

## Conflict of Interest Statement

The authors declare that the research was conducted in the absence of any commercial or financial relationships that could be construed as a potential conflict of interest.

## References

[1] STATISTIK_AUSTRIA. Spitalsentlassungen 2010 Aus Akutkrankenanstalten nach Hauptdiagnosen, Alter, Geschlecht und Aufenthaltsdauer und Standort der Krankenanstalt – Österreich und Bundesländer ICD-Shortlist 128 Positionen) 2012. (2012). Available from: www.statistik.at/web_de/static/spitalsentlassungen_2010_aus_akutkrankenanstalten_nach_hauptdiagnosen_alte_022088.xlsx

[2] Amid PK. Groin hernia repair: open techniques. World J Surg (2005) 29(8):1046–51. 10.1007/s00268-005-7967-x 15983714

[3] Amid PK Shulman AG Lichtenstein IL. The Lichtenstein open “tension-free” mesh repair of inguinal hernias. Surg Today (1995) 25(7):619–25. 10.1007/BF00311436 7549274

[4] Amid PK. Lichtenstein tension-free hernioplasty: its inception, evolution, and principles. Hernia (2004) 8(1):1–7. 10.1007/s10029-003-0160-y 14505236

[5] Bay-Nielsen M Kehlet H Strand L Malmstrøm J Andersen FH Wara P Quality assessment of 26,304 herniorrhaphies in Denmark: a prospective nationwide study. Lancet (2001) 358(9288):1124–8. 10.1016/S0140-6736(01)06251-1 11597665

[6] Fortelny RH Schwab R Glaser KS Puchner KU May C König F The assessment of quality of life in a trial on lightweight mesh fixation with fibrin sealant in transabdominal preperitoneal hernia repair. Hernia (2008) 12(5):499–505. 10.1007/s10029-008-0365-1 18392910

[7] Hinrichs-Rocker A Schulz K Järvinen I Lefering R Simanski C Neugebauer EA. Psychosocial predictors and correlates for chronic post-surgical pain (CPSP) – a systematic review. Eur J Pain (2009) 13(7):719–30. 10.1016/j.ejpain.2008.07.015 18952472

[8] Matthews RD Anthony T Kim LT Wang J Fitzgibbons RJ Jr Giobbie-Hurder A Factors associated with postoperative complications and hernia recurrence for patients undergoing inguinal hernia repair: a report from the VA Cooperative Hernia Study Group. Am J Surg (2007) 194(5):611–7 10.1016/j.amjsurg.2007.07.018 17936422

[9] Aasvang EK Møhl B Bay-Nielsen M Kehlet H. Pain related sexual dysfunction after inguinal herniorrhaphy. Pain (2006) 122(3):258–63. 10.1016/j.pain.2006.01.035 16545910

[10] Fortelny RH Petter-Puchner AH May C Jaksch W Benesch T Khakpour Z The impact of atraumatic fibrin sealant vs. staple mesh fixation in TAPP hernia repair on chronic pain and quality of life: results of a randomized controlled study. Surg Endosc (2012) 26(1):249–54. 10.1007/s00464-011-1862-3 21853390

[11] Pokorny H Klingler A Schmid T Fortelny R Hollinsky C Kawji R Recurrence and complications after laparoscopic versus open inguinal hernia repair: results of a prospective randomized multicenter trial. Hernia (2008) 12(4):385–9 10.1007/s10029-008-0357-1 18283518

[12] Butters M Redecke J Koninger J. Long-term results of a randomized clinical trial of Shouldice, Lichtenstein and transabdominal preperitoneal hernia repairs. Br J Surg (2007) 94(5):562–5. 10.1002/bjs.5733 17443855

[13] Poobalan AS Bruce J King PM Chambers WA Krukowski ZH Smith WC. Chronic pain and quality of life following open inguinal hernia repair. Br J Surg (2001) 88(8):1122–6. 10.1046/j.0007-1323.2001.01828.x 11488800

[14] Bay-Nielsen M Perkins FM Kehlet H. Pain and functional impairment 1 year after inguinal herniorrhaphy: a nationwide questionnaire study. Ann Surg (2001) 233(1):1–7. 10.1097/00000658-200101000-00001 11141218 PMC1421158

[15] Callesen T Bech K Kehlet H. Prospective study of chronic pain after groin hernia repair. Br J Surg (1999) 86(12):1528–31. 10.1046/j.1365-2168.1999.01320.x 10594500

[16] Bright E Reddy VM Wallace D Garcea G Dennison AR. The incidence and success of treatment for severe chronic groin pain after open, transabdominal preperitoneal, and totally extraperitoneal hernia repair. World J Surg (2010) 34(4):692–6. 10.1007/s00268-010-0410-y 20130871

[17] Ferzli GS Edwards ED Khoury GE Chronic pain after inguinal herniorrhaphy. J Am Coll Surg (2007) 205(2):333–41 10.1016/j.jamcollsurg.2007.02.081 17660082

[18] Poobalan AS Bruce J Smith WC King PM Krukowski ZH Chambers WA A review of chronic pain after inguinal herniorrhaphy. Clin J Pain (2003) 19(1):48–54 10.1097/00002508-200301000-00006 12514456

[19] O’Dwyer PJ Kingsnorth AN Molloy RG Small PK Lammers B Horeyseck G. Randomized clinical trial assessing impact of a lightweight or heavyweight mesh on chronic pain after inguinal hernia repair. Br J Surg (2005) 92(2):166–70. 10.1002/bjs.4833 15584057

[20] Amid PK Shulman AG Lichtenstein IL Hakakha M. Biomaterials for abdominal wall hernia surgery and principles of their applications. Langenbecks Arch Chir (1994) 379(3):168–71. 10.1007/BF00680113 8052058

[21] Weyhe D Schmitz I Belyaev O Grabs R Müller KM Uhl W Experimental comparison of monofile light and heavy polypropylene meshes: less weight does not mean less biological response. World J Surg (2006) 30(8):1586–91 10.1007/s00268-005-0601-0 16855805

[22] Engelsman AF van Dam GM van der Mei HC Busscher HJ Ploeg RJ. In vivo evaluation of bacterial infection involving morphologically different surgical meshes. Ann Surg (2010) 251(1):133–7. 10.1097/SLA.0b013e3181b61d9a 19864938

[23] Bringman S Wollert S Osterberg J Smedberg S Granlund H Heikkinen TJ. Three-year results of a randomized clinical trial of lightweight or standard polypropylene mesh in Lichtenstein repair of primary inguinal hernia. Br J Surg (2006) 93(9):1056–9. 10.1002/bjs.5403 16862613

[24] Descottes B Bagot d’Arc M Fibrin sealant in inguinal hernioplasty: an observational multicentre study in 1,201 patients. Hernia (2009) 13(5):505–10 10.1007/s10029-009-0524-z 19590820 PMC2759023

[25] Lovisetto F Zonta S Rota E Mazzilli M Bardone M Bottero L Use of human fibrin glue (Tissucol) versus staples for mesh fixation in laparoscopic transabdominal preperitoneal hernioplasty: a prospective, randomized study. Ann Surg (2007) 245(2):222–31 10.1097/01.sla.0000245832.59478.c6 17245175 PMC1876985

[26] Martín-Cartes JA Morales-Conde S Suárez-Grau JM Bustos-Jiménez M Cadet-Dussort JM López-Bernal F Role of fibrin glue in the prevention of peritoneal adhesions in ventral hernia repair. Surg Today (2008) 38(2):135–40. 10.1007/s00595-007-3590-9 18239870

[27] Schug-Pass C Lippert H Kockerling F. Mesh fixation with fibrin glue (Tissucol/Tisseel(R)) in hernia repair dependent on the mesh structure-is there an optimum fibrin-mesh combination?-Investigations on a biomechanical model. Langenbecks Arch Surg (2010) 395(5):569–74. 10.1007/s00423-009-0466-z 19184090

[28] Fortelny RH Petter-Puchner AH Glaser KS Redl H. Use of fibrin sealant (Tisseel/Tissucol) in hernia repair: a systematic review. Surg Endosc (2012) 26(7):1803–12. 10.1007/s00464-012-2156-0 22278103

[29] Petersen S Henke G Freitag M Faulhaber A Ludwig K. Deep prosthesis infection in incisional hernia repair: predictive factors and clinical outcome. Eur J Surg (2001) 167(6):453–7. 10.1080/110241501750243815 11471671

[30] Kuo YC Mondschein JI Soulen MC Patel AA Nemeth A Stavropoulos SW Drainage of collections associated with hernia mesh: is it worthwhile? J Vasc Interv Radiol (2010) 21(3):362–6. 10.1016/j.jvir.2009.11.009 20171558

[31] Petter-Puchner AH Walder N Redl H Schwab R Ohlinger W Gruber-Blum S Fibrin sealant (Tissucol) enhances tissue integration of condensed polytetrafluoroethylene meshes and reduces early adhesion formation in experimental intraabdominal peritoneal onlay mesh repair. J Surg Res (2008) 150(2):190–5. 10.1016/j.jss.2007.12.796 18468639

[32] Campanelli G Pascual MH Hoeferlin A Rosenberg J Champault G Kingsnorth A Randomized, controlled, blinded trial of tisseel/tissucol for mesh fixation in patients undergoing Lichtenstein technique for primary inguinal hernia repair: results of the TIMELI trial. Ann Surg (2012) 255(4):650–7. 10.1097/SLA.0b013e31824b32bf 22395092

[33] McCarthy M Jr Chang CH Pickard AS Giobbie-Hurder A Price DD Jonasson O Visual analog scales for assessing surgical pain. J Am Coll Surg (2005) 201(2):245–52. 10.1016/j.jamcollsurg.2005.03.034 16038823

